# The impact of thickness and thermal annealing on refractive index for aluminum oxide thin films deposited by atomic layer deposition

**DOI:** 10.1186/s11671-015-0757-y

**Published:** 2015-02-06

**Authors:** Zi-Yi Wang, Rong-Jun Zhang, Hong-Liang Lu, Xin Chen, Yan Sun, Yun Zhang, Yan-Feng Wei, Ji-Ping Xu, Song-You Wang, Yu-Xiang Zheng, Liang-Yao Chen

**Affiliations:** Shanghai Engineering Research Center of Ultra-Precision Optical Manufacturing, Department of Optical Science and Engineering, Laboratory of Micro and Nano Photonic Structures, Ministry of Education, Fudan University, Shanghai, 200433 China; State Key Laboratory of ASIC & System, Fudan University, Shanghai, 200433 China; National Laboratory for Infrared Physics, Shanghai Institute of Technical Physics, Chinese Academy of Sciences, Shanghai, 200083 China; Key Laboratory of Infrared Image Materials and Devices, Shanghai Institute of Technical Physics, Chinese Academy of Sciences, Shanghai, 200083 China

**Keywords:** ALD, Al_2_O_3_ thin film, Optical properties, Spectroscopic ellipsometry

## Abstract

The aluminum oxide (Al_2_O_3_) thin films with various thicknesses under 50 nm were deposited by atomic layer deposition (ALD) on silicon substrate. The surface topography investigated by atomic force microscopy (AFM) revealed that the samples were smooth and crack-free. The ellipsometric spectra of Al_2_O_3_ thin films were measured and analyzed before and after annealing in nitrogen condition in the wavelength range from 250 to 1,000 nm, respectively. The refractive index of Al_2_O_3_ thin films was described by Cauchy model and the ellipsometric spectra data were fitted to a five-medium model consisting of Si substrate/SiO_2_ layer/Al_2_O_3_ layer/surface roughness/air ambient structure. It is found that the refractive index of Al_2_O_3_ thin films decrease with increasing film thickness and the changing trend revised after annealing. The phenomenon is believed to arise from the mechanical stress in ALD-Al_2_O_3_ thin films. A thickness transition is also found by transmission electron microscopy (TEM) and SE after 900°C annealing.

## Background

Aluminum oxide (Al_2_O_3_) thin films are used as gate dielectric films in electronic devices [1], protective coating layer in magnetic read heads [2], encapsulation layer in light emitting diodes [3], antireflection coating in solar thermal cells [4], and many other areas [5-7]. These applications benefit from the excellent optical and electrical properties of Al_2_O_3_ films such as wide bandgap, high conduction, high compatibility with Si substrate, and high dielectric constant [8]. The properties of Al_2_O_3_ films have been studied a lot [9-11]. However, most articles focused on electrical and mechanical properties of Al_2_O_3_ films. The research on optical properties of Al_2_O_3_ films_,_ especially for Al_2_O_3_ films thinner than 50 nm, is still lacking. The applications of optical critical dimension, *in situ* spectroscopic ellipsometry (SE) and phase measurements in inspection are widely used in semiconductor process and solar cells. These applications are dependent on the accuracy of dielectric constants. The inaccurate optical constant of Al_2_O_3_ can introduce errors in fabricating procedure and further influence the performance of devices. So the study of optical properties of Al_2_O_3_ thin films is needed.

Atomic layer deposition (ALD) is one of the most popular chemical vapor deposition methods used in oxide film fabrication [12]. For its low temperature and monolayer deposition, Al_2_O_3_ ultrathin films with a smooth and defect-free surface can be deposited. Therefore, ALD-Al_2_O_3_ films are widely used in recent researches [13,14].

SE is routinely used in optical characterization and film thickness determination. In the SE measurement, a linearly polarized light is illuminated on the sample. The polarization state will be changed after the light reflected. Two parameters, the amplitude ratio (*Ψ*) and phase shift (*Δ*) between reflected *p-* and *s-*polarized light, are obtained from the measurement [15]. The ellipsometric spectra can be fitted to the optical model based on the film structure, then the optical properties and film thickness of the measured material can be revealed [16-18]. Its noncontact, nondestructive characteristics are ideal for many situations when film thickness or dielectric constants are needed [19,20].

In this paper, the thickness dependence of refractive index for ALD-Al_2_O_3_ films is investigated by SE. An anomaly change trend of refractive index for Al_2_O_3_ films was reported. The changing trend reversed after the Al_2_O_3_ films were annealed in nitrogen condition at different temperatures. The thickness transition was observed through transmission electron microscopy (TEM) and SE. The change of dielectric constant was explained by the changing of dielectric polarization after annealing.

## Methods

The Al_2_O_3_ films were deposited by a thermal ALD reactor (Picosun R-series, Espoo, Finland) on Si substrate. Trimethylaluminum (TMA; Al(CH_3_)_3_) and water (H_2_O) were used as metal and oxidation precursors, respectively. The reacting temperature is 100°C. The characteristic analysis of surface morphology was performed by atomic force microscopy (AFM; Bruker Dimension Icon VT-1000, Santa Barbara, CA, USA) in tapping mode. The ellipsometric spectra were measured by a SE system (J.A. Woollam Co. M2000X-FB-300XTF, Lincoln, NE, USA) over the wavelength range of 250 to 1,000 nm at incident angle of 65°. The thickness of SiO_2_ and Al_2_O_3_ layers were identified by TEM (FEI Tecnai G2 F20, Hillsboro, OR, USA), Then the ALD-Al_2_O_3_ samples were cut into three pieces and annealed at 400°C, 600°C, and 900°C in nitrogen atmosphere. A rapid thermal process system (RTP; AS-ONE, Montpellier, France) was used. The annealed samples were researched by AFM, TEM, and SE again to perform further analysis.

**Figure 1:**
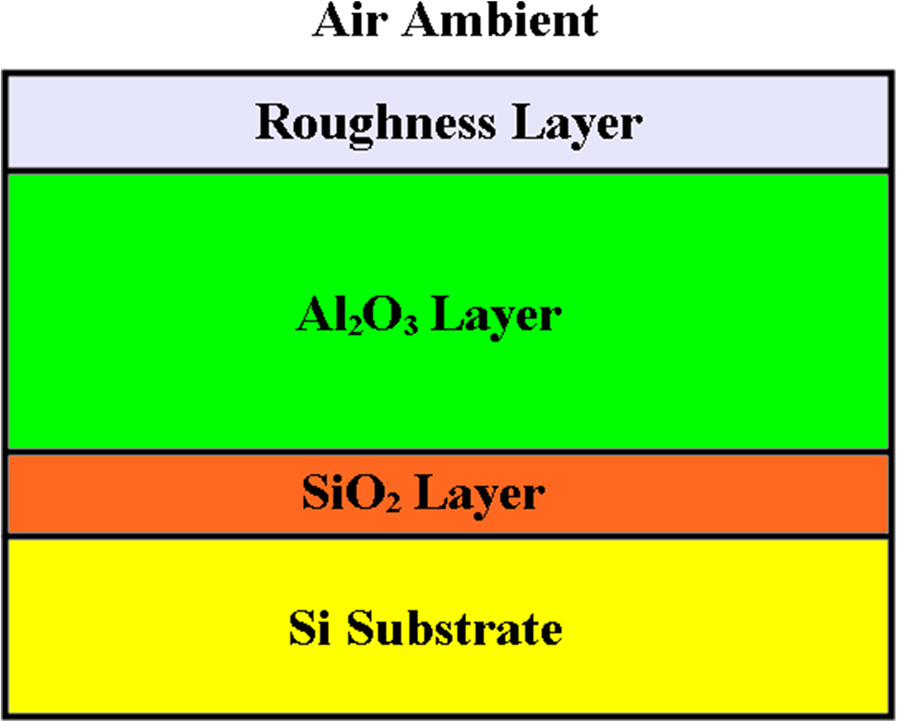
**The schematic of optical model used in SE fitting for Al**_2_ **O**_3_ **thin films.**

Considering the Si substrate always have a native oxide layer [21], the ellipsometric spectra were collected for the Si substrate with oxide layer and ALD-Al_2_O_3_ thin film, respectively. The RMS roughness obtained from AFM helps determining the thickness of roughness layer. So the optical model used in SE fitting is Si substrate/SiO_2_ layer/Al_2_O_3_ layer/surface roughness/air ambient, as shown in Figure 1. The dispersion model of Al_2_O_3_ used in SE fitting is Cauchy model [22].

## Results and discussion

**Figure 2:**
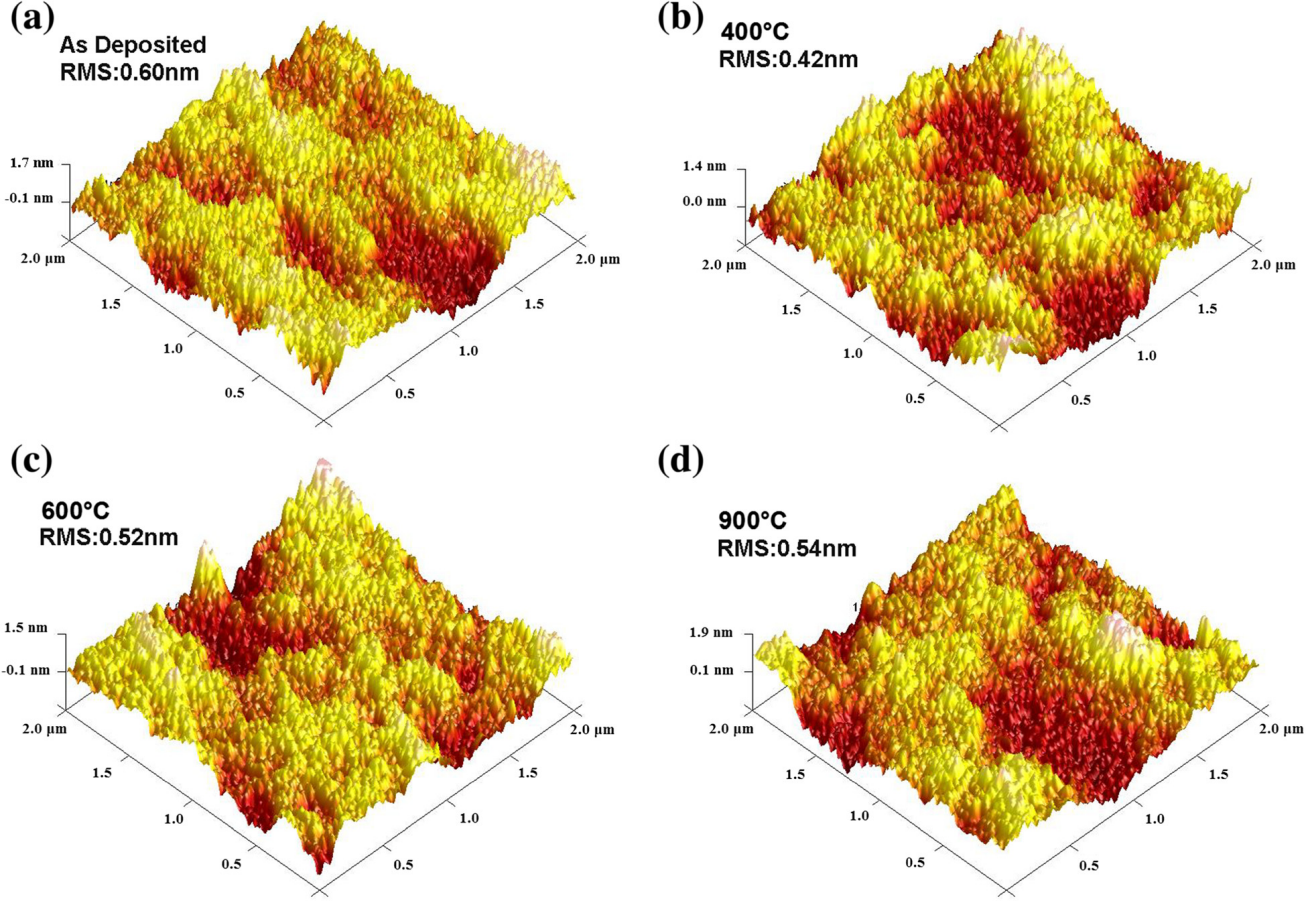
**AFM images of 500 cycles Al**_2_ **O**_3_ **film. (a)** As deposited and annealed at **(b)** 400°C, **(c)** 600°C, and **(d)** 900°C.

The numbers of ALD cycles in the deposition were 50, 100, 300, and 500. Figure 2 shows the AFM images of selected ALD-Al_2_O_3_ thin film. The surface of the samples is smooth and crack-free, which indicates that Al_2_O_3_ films were well fabricated. The root mean square roughness (RMS roughness) information of all samples is listed in Table 1. The thickness of surface roughness layer used in SE fitting is fixed as the RMS value. And the roughness layer is described by a Bruggeman effective medium approximation mixed by 50% Al_2_O_3_ and 50% void [23].

**Table 1:** **RMS roughness of ALD-Al**_2_ **O**_3_ **thin films**

	**Sample (cycles)**			
	**50**	**100**	**300**	**500**
**Annealing temperature (°C)**	**RMS roughness (nm)**			
As deposited	0.65	0.62	0.49	0.60
400	0.54	0.55	0.58	0.42
600	0.49	0.51	0.53	0.52
900	0.54	0.46	0.40	0.54

**Figure 3:**
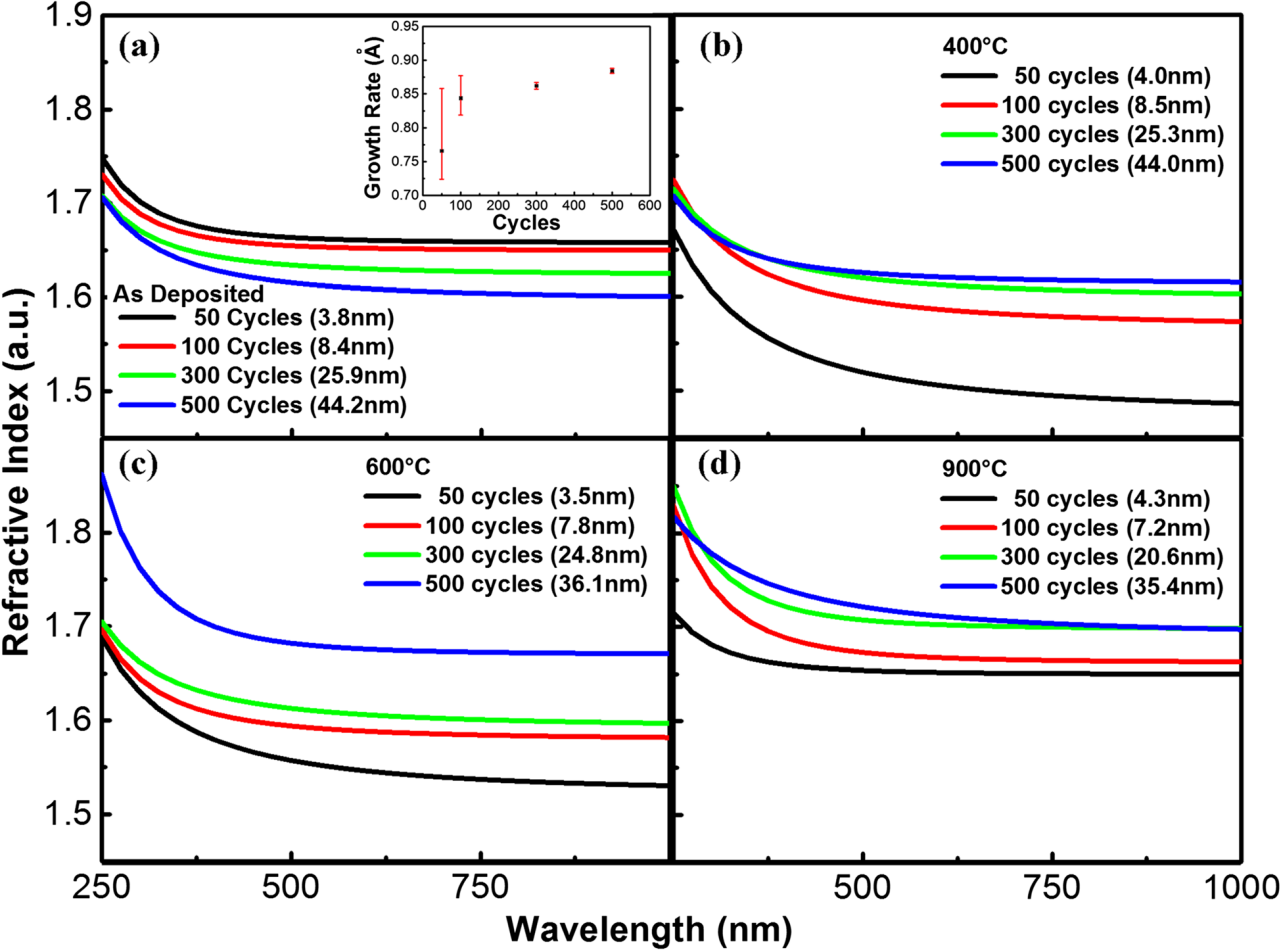
**Thickness dependence of refractive index for ALD-Al**_2_ **O**_3_ **films. (a)** As deposited and annealed at **(b)** 400°C, **(c)** 600°C, and **(d)** 900°C in nitrogen. The inset is the growth rate of as deposited Al_2_O_3_ films.

Considering Al_2_O_3_ is transparent in visible region, the optical model of Al_2_O_3_ used in SE fitting is Cauchy model, which is defined as follows [22]:

1 $$ n\left(\lambda \right)=A+\frac{B}{\lambda^2}+\frac{C}{\lambda^4} $$

2 $$ k=0 $$

where *A*, *B*, and *C* are the material coeffients that define the real part of the refractive index *n*(*λ*). Figure 3a illustrates the thickness dependence of refractive index for as deposited ALD-Al_2_O_3_ films revealed by SE fitting. It can be found that with increasing thickness, the refractive index of Al_2_O_3_ is decreasing, which is contrary to Al_2_O_3_ films thicker than 50 nm [24-26]. From the inset of Figure 3a, we can know that the Al_2_O_3_ films were grown at a speed of 0.88 Å/cycle. The growth rate becomes stable when ALD cycle is higher than 100.

**Figure 4:**
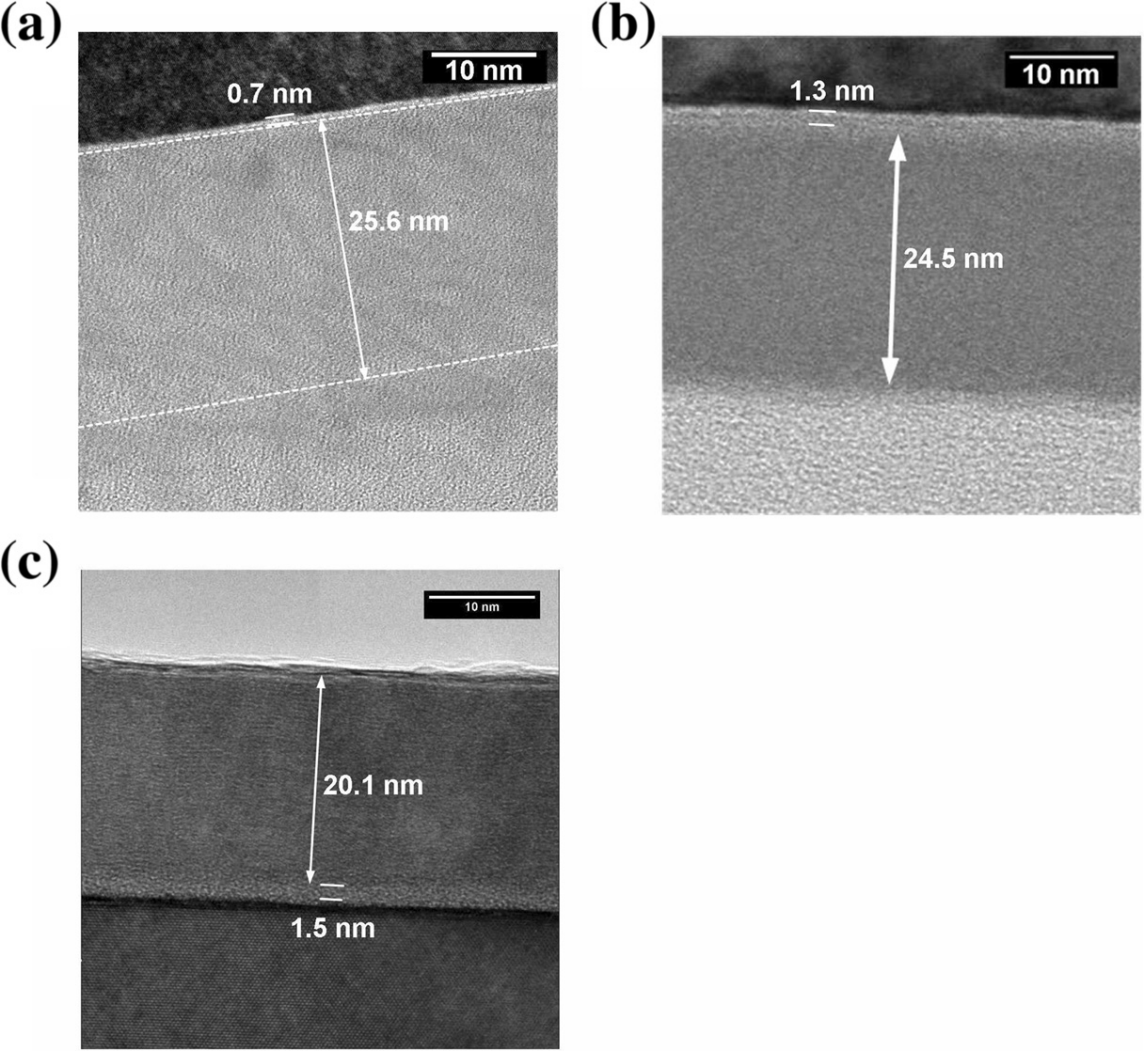
**TEM pictures of 300 cycles of Al**_2_ **O**_3_ **film. (a)** As deposited and annealed at **(b)** 600°C and **(c)** 900°C.

The Al_2_O_3_ thin films were then annealed at 400°C, 600°C, and 900°C, respectively. The changing trend of refractive index at each annealing temperature is illustrated in Figure 3b,c,d. It is indicated that the thickness dependence of refractive index for Al_2_O_3_ films reversed and shows regular evolution rule after annealing. Furthermore, the thicknesses of Al_2_O_3_ films show a significant decrease after 900°C annealing. TEM pictures in Figure 4 also support the SE results. The thickness of SiO_2_, 300 cycles of Al_2_O_3_, and RMS layer at different annealing temperatures in SE fitting and TEM measurements are compared in Table 2. The thickness of SiO_2_ layer slightly increased after annealing. And the Al_2_O_3_ film went through a densification process after annealing.

**Table 2:** **Thickness comparison between SE and TEM on 300 cycles Al**_2_ **O**_3_ **film**

	**Thickness (nm)**					
	**SE**			**TEM**		
**Annealing temperature (°C)**	**SiO**_2_	**Al**_2_ **O**_3_	**RMS**	**Al**_2_ **O**_3_	**SiO**_2_	**RMS**
As deposited	1.0	25.9	0.49	0.7	25.6	-
600	1.0	24.8	0.53	1.3	24.5	-
900	1.0	20.6	0.40	1.5	20.2	-

Generally, the ALD-Al_2_O_3_ film will be under a stress state after it is deposited. And for thin films under 50 nm, the effect of internal stress is strongly related to thickness [27,28]. The anomaly changing trend of refractive index for Al_2_O_3_ thin films is only observed in the as deposited samples. The reverse of changing trend may be contributed to two reasons caused by annealing process: stress release and phase transition.

**Figure 5:**
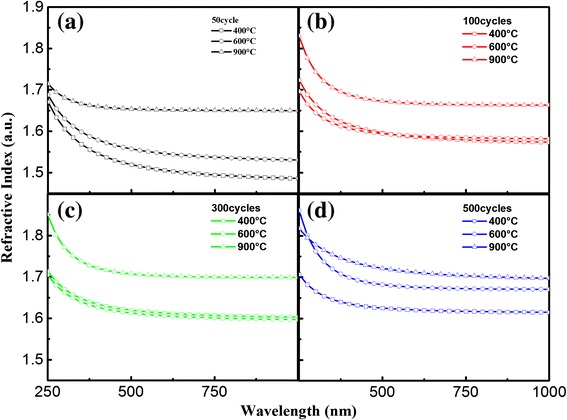
**Annealing temperature dependence of refractive index for ALD-Al**_2_ **O**_3_ **with different thicknesses. (a)** 50 cycles, **(b)** 100 cycles, **(c)** 300 cycles, and **(d)** 500 cycles.

To further understand the effect of annealing process, the refractive index depending on annealing temperature for each sample are researched and given in Figure 5. A significant increase in refractive index after 900°C annealing can be noted. This variation can also be observed from the thickness decreasing illustrated in Figures 3 and 4, which means the films are more compact or may become crystallized. But the crystal grain is not observed from TEM pictures in Figure 4b,c. The Al_2_O_3_ thin films are not crystallized after annealing. The transition of the films is due to stress release and densification caused by annealing.

The refractive index of Al_2_O_3_ films increased after thickness transition. This phenomenon can be explained by classical dielectric theory. For a transparent material, the dielectric constant *ε* is given by [22].

3 $$ \varepsilon =1+\frac{P}{\varepsilon_0E}={n}^2 $$

4 $$ P={\displaystyle \sum_i}{q}_i{l}_i $$

where *P* is the dielectric polarization, *n* is the refractive index, *ε*_0_ is the free-space permittivity, and *E* is the electric field. The *q*_i_ and *l*_i_ are electric charge of electric dipole and distance between the charge pair, respectively. So the dielectric polarization is related to the electric charge and distances of dipoles in the material. The dielectric constant will become larger if the dielectric polarization is larger.

In the process of annealing, the vacancies are filled during annealing and the thickness of Al_2_O_3_ films will decrease. The charge of electric dipole is then increased and leads to a higher dielectric polarization. This is often accompanied with a decreasing of total binding energy [29], which agrees with previous reports on Al_2_O_3_ films [10,27].

The annealing process, which is believed as an efficient method to release the stress or leads to a thickness transition, turned the changing trend of refractive index back to normal. The stress in ALD-Al_2_O_3_ thin films caused the anomaly trend. And the stress has been released after a 400°C annealing. A higher annealing temperature further led to a thickness transition of Al_2_O_3_ films. Both stress release and thickness transition will have a significant influence on the refractive index of Al_2_O_3_ films.

## Conclusions

In summary, the ALD-Al_2_O_3_ thin films with various thicknesses were fabricated and annealed at different temperatures. The AFM measurement indicated that the surface roughness of Al_2_O_3_ thin films was less than 1 nm. The SE analysis revealed that the refractive index of as deposited Al_2_O_3_ thin films decreases with increasing film thickness. And this anomaly phenomenon disappeared after annealing. Further analysis on SE and TEM data shows that the stress of as deposited Al_2_O_3_ caused the anomaly changing trend of refractive index. And the refractive index becomes higher after 900°C annealing, which is contributed by vacancy filling induced higher dielectric polarization. The revolution of optical constant will affect other properties of Al_2_O_3_ thin films and leads to new features. The results given in this work will be helpful in further fabrication and application of Al_2_O_3_ thin films.

## Abbreviations

AFM: atomic force microscopy
ALD: atomic layer deposition
RMS roughness: root mean square roughness
SE: spectroscopic ellipsometry
TEM: transmission electron microscopy
